# Prevalence and factors associated with the presence of oral infections in pregnant women in a medium-sized municipality in Brazil

**DOI:** 10.1590/1414-431X2025e14436

**Published:** 2025-05-30

**Authors:** A.P.N. Godoi, G.C.S. Bernardes, L.N. Godoi, L.S. Nogueira, G.M. Rocha, M. Barros-Pinheiro

**Affiliations:** 1 Universidade Federal de São João del-Rei, Campus Centro-Oeste Dona Lindu, Divinópolis, MG, Brasil; 2 Departamento de Análises Clínicas e Toxicológicas, Faculdade de Ciências Farmacêuticas, Universidade Federal de Alfenas, Alfenas, MG, Brasil

**Keywords:** Pregnancy, Prenatal, Dental care, Pregnancy complications, Infections

## Abstract

The objective of this study was to estimate the prevalence and factors associated with oral changes, including infections and other oral conditions in pregnant women who underwent prenatal care in public health units in the city of Divinópolis, Minas Gerais. This was a cross-sectional study carried out with baseline data from a cohort of 588 pregnant women (aged 14 to 43 years) from 2019 to 2023. Data were collected through interviews and oral clinical examination, using a structured questionnaire. The event of interest was the presence of oral infection. The absolute and relative frequencies of the variables were calculated. Logistic regression was used to evaluate the association between the explanatory variables and the presence of oral infection, with estimation of odds ratios and 95% confidence intervals. Among the participants, 47.8% had oral infections. In the multivariate analysis, the variables that showed a significant association with oral infection were: lower education, last visit to the dentist more than 6 months ago, lower frequency of flossing, regular intake of alcoholic beverages, and self-report of poor oral health. This study highlighted sociodemographic and behavioral disparities and the importance of prenatal dental care. Insights for adapting interventions were provided, although further studies are needed.

## Introduction

During pregnancy, oral changes play a crucial role not only for the health of the oral cavity, but also for the maternal and fetal health. Pregnancy induces hormonal changes that can predispose pregnant women to the exacerbation of conditions such as periodontal diseases and caries, increasing the risk of obstetric complications. Oral infections have been linked to premature births and low birth weight babies. Therefore, prenatal dental care transcends aesthetics, being an essential component for promoting a healthy pregnancy and the well-being of the maternal-fetal binomial (1).

In 2022 in the municipality of Divinópolis, Minas Gerais (MG), there were 2,445 live births (taking into account maternal residence), 339 of which were born prematurely. Of the live births, four pregnant women did not undergo any prenatal consultation. In 2021, there were 2,601 live births in the city and seven maternal deaths (during pregnancy, childbirth, and/or up to 1 year postpartum) (2), resulting in a maternal death rate of 269 deaths per 100,000 live births. This rate was more than double the Brazilian average, which was 113.02 maternal deaths per 100,000 live births (3). In August 2023, the National Health Council recommended the expansion of measures to combat maternal mortality to the Ministry of Health that were established by the World Health Organization in 2015 through the Global Compact, which consists of several sustainable development objectives, covering the 193 countries that make up its members. One of the objectives is that by 2030, its members must seek to reduce the maternal mortality rate to below 70 deaths for every 100,000 live births. According to the Pan American Health Organization (Organização Pan-Americana de Saúde - OPAS) (4), the condition of maternal mortality in Latin America and the Caribbean is alarming: approximately 8,000 women lose their lives annually due to complications associated with pregnancy; disparities in socioeconomic status, gender, ethnicity, education, place of residence, and age increase the risk of death during pregnancy, childbirth, or in the postpartum period; nine out of ten maternal deaths can be prevented with timely access to evidence-based maternal care, including contraceptive methods; and the COVID-19 pandemic caused a two-decade setback in maternal health rates.

According to the Ministry of Health (5), prenatal care aims to monitor and carry out interventions that can improve the health condition of the mother and fetus, resulting in the healthy birth of a newborn, with no impact on maternal health, including psychosocial aspects and educational and preventive activities. In this context, health units should be the preferred point of admission for pregnant women into the health system, as they are a strategic place of care to maximize the reception of these women.

During prenatal care, in addition to medical monitoring, it is important to offer specific dental care. This assistance should prevent and treat oral health problems that occur during pregnancy and promote the health and quality of life of the pregnant woman and her baby. Ideally, every woman should have this information and access to preventive dental care from childhood. This could reduce the need for invasive dental procedures and possible complications, such as pain, inflammation, and oral infections that could disrupt this important moment in their lives (6).

Therefore, the objective of this study was to evaluate the performance of prenatal dental care and estimate the prevalence of and factors associated with oral changes, including infections and other oral conditions, in pregnant women who underwent prenatal care in health units in Divinópolis from September 2019 to November 2023.

## Material and Methods

### Study design and participants

This was a cross-sectional study with baseline data from a cohort. The inclusion criteria were pregnant women of any age who underwent prenatal consultations in basic health units in Divinópolis, during the study period. Pregnant women who did not have minimal autonomy (due to mental or conscious changes), according to the interviewer's subjective assessment, who did not reside in the municipality, and who would not continue with prenatal care in the Public Health System (Sistema Único de Saúde - SUS), as well as those who did not have any record of prenatal consultations in their medical records were excluded. The study was approved by the UFSJ Research Ethics Committee through opinion 3.614.386, CAEE 20648719.3.0000.5545.

### Procedures and data collection

The sample size was calculated, pre-tests were conducted, researchers were trained and evaluated, the instrument was validated, and a pilot study was performed. The questionnaire used in the semi-structured interview, along with the informed consent form and assent form, was specifically designed for this study. Training sessions were conducted with the researchers both before and after the pre-test and pilot study.

The pre-test involved eight pregnant women from the private health network who voluntarily agreed to participate. Based on the pre-test results, adjustments were made to the questionnaire. Subsequently, a pilot study was conducted with fifteen pregnant women from a single health unit. The participants in the pre-test and pilot study were not included in the final research population.

For data collection, pregnant women were approached in the waiting room of their antenatal appointments and received basic information about the study objectives. Those who agreed to participate signed the informed consent form in a reserved area. The participants then underwent a semi-structured, face-to-face interview, followed by a clinical evaluation.

The clinical evaluation, performed by a single trained examiner, included an inspection of the oral cavity and the completion of an odontogram and clinical data form. This intraoral assessment was conducted visually under standardized artificial light (portable focus lamp) using a flat intraoral mirror. The women were seated on a regular chair during the examination.

Additionally, data from medical records were obtained by reviewing both physical and electronic prenatal consultation records maintained in the data systems of Divinópolis City Hall.

The survey data were obtained based on a sampling plan designed for a population of 2,600 new pregnant women annually in Divinópolis, across all age groups. The expected frequency of oral changes was estimated at 35%, with a 95% confidence level and a design effect of 1.0. The total calculated sample size was 374 participants, and considering an estimated 15% loss, the aim was to recruit at least 431 pregnant women during the data collection period.

All basic health units in Divinópolis were included in the study. However, data collection occurred at different times, as prenatal care was unavailable in some units during certain periods.

### Study variables

The presence of dental calculus, gingivitis, gingival bleeding whether spontaneous or to the touch, periodontitis, periodontal abscess, gingival abscess, diastemas, crowding, changes in adjacent soft tissues, melasma, and other unspecified oral changes were verified, as was the use of removable or fixed prostheses and orthodontic appliances. Furthermore, oral hygiene was classified as poor (visible bacterial plaque, dental calculus, and food debris), fair (visible bacterial plaque and dental calculus); good (visible bacterial plaque), or excellent (absence of visible bacterial plaque).

The response variable was the presence of oral infection, defined by the detection of any infectious process in the soft or hard tissues of the oral cavity during clinical evaluation. The explanatory variables were dichotomized and evaluated according to the following criteria: a) sociodemographic characteristics: age, color, marital status, education, occupation, and family income; b) behavioral characteristics: regular use of tobacco, regular use of alcoholic beverages, regular use of illicit drugs, daily consumption of sweets and sugary foods, daily consumption of bread, pasta, or tubers, daily consumption of processed foods, regular physical activity during pregnancy; c) clinical and oral health-related characteristics: prenatal classification, history of previous pregnancies, comorbidities, use of medications, gestational age, being recommended to see a dentist during pregnancy, knowing that pregnant women can have dental treatment, having noticed changes in the mouth after pregnancy, knowing that oral health can affect pregnancy, considering it necessary to go to the dentist during pregnancy, having knowledge about prenatal dental care, use of a toothbrush, use of dental floss, and use of toothpicks.

### Data analysis

The absolute and relative frequencies of the variables of interest were calculated. The association between explanatory variables and the response variable was evaluated using contingency tables with chi-squared tests, with a significance level of 0.05. The magnitude of the association was assessed by estimating the odds ratio (OR), with a 95% confidence interval (95%CI). Next, multivariate analysis was performed using multiple logistic regression. To build the initial model, variables that had a P-value of less than 0.20 in the bivariate analysis were included. After the sequential exclusion of adjusted variables with a non-significant association, only variables with a P-value lower than 0.05 remained in the final model. Data were analyzed using the EpiInfo^®^ version 7.2.2.6 for Windows program.

## Results

During the collection period, a total of 982 pregnant women were approached, 205 (20.9%) refused to participate and 189 (19.2%) were excluded from the research (115 for not having data in their medical records, 71 for not having pre-natal care in the Public Health System (SUS), and three for not having undergone a clinical oral examination). Therefore, the final sample included 588 (59.9%) pregnant women in the study.

The majority (82.6%) of participants were between 18 and 34 years of age (with an average age of 27.3 years), were married or in a stable relationship (60.5%), had a paid occupation (56.9%), and had attended high school or higher (79.2%). The smallest proportion of participants were white (31.7%), were classified as high-risk pregnant women (31.9%), and had not had a dental appointment in the previous six months (43.6%). Regarding oral hygiene, the majority of participants brushed their teeth twice a day or more (94.1%), used dental floss once a day or less (75.5%), and used toothpicks once a day or less (89.8%). Furthermore, almost 89.5% of pregnant women reported not knowing what prenatal dental care is. The majority consumed alcohol regularly (65.1%) and few used tobacco (25.8%) or illicit drugs (9.2%) regularly. A high proportion of participants reported daily consumption of sweets (49.9%), bread, pasta, or tubers (79.4%), and processed foods (84.6%) (Table 1).

**Table 1:** Sociodemographic, behavioral, clinical, and oral health characteristics of pregnant women (n=588).

Variables	n (%)
**Sociodemographic characteristics**	
Age (years)	
18-34	486 (82.6)
14-17 and 35 or more	102 (17.3)
Self-declared race	
White	186 (31.7)
Non-white	401 (68.3)
Marital status	
Common law/married	356 (60.5)
Others	232 (39.5)
Schooling	
High school or higher	465 (79.2)
Up to complete primary education	122 (20.8)
Occupation	
Formally employed/self-employed	334 (56.9)
Domestic chores	253 (43.1)
Family income	
More than 2 minimum salaries	95 (17.9)
Up to 2 minimum salaries	436 (82.1)
**Behavioral characteristics**	
Regular tobacco use	
Yes	151 (25.8)
No	435 (74.2)
Regular use of alcoholic beverages	
Yes	375 (65.1)
No	201 (34.9)
Regular use of illicit drugs	
Yes	54 (9.2)
No	533 (90.8)
Regular physical activity during pregnancy	
Yes	71 (12.1)
No	514 (87.9)
Daily consumption of sweets and sugary foods	
Yes	280 (49.9)
No	281 (50.1)
Daily consumption of bread, pasta, or tubers	
No	115 (20.6)
Yes	443 (79.4)
Daily consumption of processed foods	
No	88 (15.4)
Yes	482 (84.6)
**Clinical and oral health-related characteristics**	
Prenatal classification	
Normal risk	211 (68.1)
High risk	99 (31.9)
History of previous pregnancies	
Yes	342 (58.5)
No	243 (41.5)
Comorbidities	
Yes	305 (51.9)
No	283 (48.1)
Medication use	
Yes	151 (25.7)
No	437 (74.3)
Gestational age (days)	
Up to 190	232 (39.5)
Above 190	356 (60.5)
Last dental appointment (months)	
Less than 6	254 (43.6)
More than 6	329 (56.4)
Self-report of poor oral health	
Yes	422 (78.6)
No	115 (21.4)
Received recommendation to see a dentist during pregnancy	
Yes	262 (44.6)
No	325 (55.4)
Knew that pregnant women can undergo dental treatment	
No	15 (2.7)
Yes	538 (97.3)
Has noticed changes in her mouth after pregnancy	
Yes	318 (54.1)
No	270 (45.9)
Knew that oral health can influence pregnancy	
Yes	425 (82.5)
No	90 (17.5)
Considers it necessary to go to the dentist during pregnancy	
No	21 (3.7)
Yes	548 (96.3)
Has knowledge about prenatal dentistry	
Yes	61 (10.5)
No	522 (89.5)
Use of a toothbrush	
2 times daily or more	553 (94.1)
1 time daily or less	35 (5.9)
Use of dental floss	
2 times daily or more	144 (24.5)
1 time daily or less	444 (75.5)
Use of a toothpick	
2 times daily or more	60 (10.2)
1 time daily or less	527 (89.8)

The most frequent oral changes were poor oral hygiene (66.3%), hard tissue infection (44.4%), gingivitis (68.4%), and the presence of dental calculus (76.9%) (Table 2).

**Table 2:** Dental changes found during oral examination of pregnant women (n=588).

Variables	n (%)
Oral hygiene	
Bad	390 (66.3)
Regular	72 (12.2)
Good	77 (13.1)
Excellent	56 (9.5)
Dental calculus	
Yes	452 (76.9)
No	136 (23.1)
Gingivitis	
Yes	402 (68.4)
No	186 (31.6)
Spontaneous gingival bleeding	
Yes	159 (27.0)
No	429 (73.0)
Gingival bleeding to the touch	
Yes	395 (67.2)
No	193 (32.8)
Periodontitis	
Yes	93 (15.8)
No	494 (84.2)
Periodontal abscess	
Yes	59 (10.1)
No	526 (89.9)
Gingival abscess	
Yes	66 (11.2)
No	521 (88.8)
Presence of diastemas	
Yes	134 (22.8)
No	454 (77.2)
Presence of crowding	
Yes	341 (58.0)
No	247 (42.0)
Changes in adjacent soft tissues	
Yes	164 (27.9)
No	424 (72.1)
Presence of melasma	
Yes	188 (32.0)
No	400 (68.0)
Other unspecified oral changes	
Yes	199 (33.8)
No	389 (66.2)
Use of removable or fixed prosthesis	
Yes	41 (7.0)
No	547 (93.0)
Use of orthodontic appliance	
Yes	129 (21.9)
No	459 (78.0)
Soft tissue infection	
Yes	149 (25.3)
No	439 (74.7)
Hard tissue oral infection	
Yes	261 (44.4)
No	327 (55.6)
Oral infection (soft and/or hard tissue)	
Yes	281 (47.8)
No	307 (52.2)

In the bivariate analysis, pregnant women whose occupation was household chores were 1.3 times more likely to have an oral infection compared than those who had a formal job. Women who had elementary education and who regularly used tobacco during pregnancy were 1.5 times more likely to have oral infections compared to those who had higher levels of education and who did not use tobacco. Pregnant women who had last visited the dentist more than 6 months ago were 1.9 times more likely to have an oral infection than those who visited the dentist less than 6 months ago. Pregnant women who reported a lack of knowledge about prenatal dental care were 1.6 times more likely to have an oral infection compared to those who reported knowledge about prenatal dental care. Pregnant women who reported using dental floss once a day or less were 1.8 times more likely to have an oral infection compared to those who reported using dental floss twice a day or more. Pregnant women who reported using toothpicks once a day or less were 3.5 times more likely to have an oral infection compared to those who reported using toothpicks twice a day or more (Table 3).

**Table 3:** Bivariate analysis of the factors associated with oral infections in pregnant women (n=588).

Variables	Oral infection (n=307)				P-value
	Total (n)	n	%	OR (95%CI)	
**Sociodemographic characteristics**					
Age (years)					
18-34	486	134	50.0	1.0	
14-17 and 35+	102	21	50.0	1.0 (0.5-1.9)	1.00
Color					
White	186	96	51.6	1.0	
Non-white	401	211	52.6	1.0 (0.7-1.5)	0.89
Marital Status					
Common law/married	356	179	50.3	1.0	
Others	232	128	55.2	1.2 (0.9-1.7)	0.28
Schooling					
High school and above	465	232	49.9	1.0	
Primary school or less	122	74	60.6	1.5 (1.0-2.3)	0.04
Occupation					
Employed/self-employed	334	164	49.1	1.0	
Unemployed	253	143	56.5	1.3 (0.9-1.9)	0.09
Family income					
Up to 2 minimum salaries	436	223	51.1	1.0	
More than 2 minimum salaries	95	46	48.4	0.90 (0.6-1.4)	0.71
**Behavioral characteristics**					
Regular smoker (tobacco)					
No	435	215	49.4	1.0	
Yes	151	90	59.6	1.5 (1.0-2.2)	0.04
Regular alcohol intake during pregnancy					
No	201	99	49.2	1.0	
Yes	375	202	53.9	1.2 (0.8-1.7)	0.33
Use of illicit drugs during pregnancy					
No	533	273	51.2	1.0	
Yes	54	33	61.1	1.5 (0.8-2.7)	0.21
Regular physical activity during pregnancy					
Yes	71	34	47.9	1.0	
No	514	270	52.5	1.2 (0.7-2.0)	0.54
Daily consumption of sweets and sugary foods					
No	281	152	54.1	1.0	
Yes	280	139	49.6	0.8 (0.6-1.2)	0.33
Consumption of bread, pasta and tubers					
No	443	237	53.5	1.0	
Yes	115	52	45.2	0.7 (0.5-1.1)	0.14
Consumption of processed foods					
No	88	52	59.1	1.0	
Yes	482	249	51.7	0.7 (0.5-1.2)	0.24
**Clinical and oral health characteristics**					
Prenatal classification					
Normal risk	391	110	70.5	1.0	
High risk	198	54	47.9	1.3 (0.5-2.11)	0.33
Knows that pregnant women can undergo dental treatment					
Yes	538	282	52.4	1.0	
No	15	07	46.7	0.8 (0.3-2.3)	0.86
Comorbidities					
No	283	137	48.4	1.0	
Yes	305	170	55.7	1.34 (0.9-1.8)	0.09
Medication use					
No	437	231	52.9	1.0	
Yes	151	76	50.3	1.1 (0.8-1.6)	0.66
Gestational Age					
Up to 190 days	232	118	50.9	1.0	
More than 190 days	356	189	53.1	1.1 (0.8-1.5)	0.66
Last visit to a dentist					
Less than 6 months	254	110	43.3	1.0	
More than 6 months	329	193	58.7	1.9 (1.3-2.6)	<0.01
Self-reported poor oral health					
No	115	78	67.8	1.0	
Yes	422	210	49.8	0.5 (0.3-0.7)	<0.01
Has received guidance on seeing a dentist during pregnancy					
No	325	182	56.0	1.0	
Yes	262	124	47.3	0.7 (0.4-0.9)	0.04
Knows that pregnant women can undergo dental treatment					
Yes	538	282	52.4	1.0	
No	15	07	46.7	0.8 (0.3-2.3)	0.86
Has noticed changes in her mouth after pregnancy					
Yes	270	129	47.8	1.0	
No	318	178	56.0	1.4 (1.0-1.9)	0.05
Oral health can influence pregnancy					
Yes	425	224	52.7	1.00	
No	90	53	58.9	1.3 (0.8-2.0)	0.34
Believes it is necessary to see a dentist during pregnancy					
No	21	09	42.9	1.00	
Yes	548	290	52.9	0.7 (0.3-1.6)	0.5
Knowledge about prenatal care					
Yes	61	26	42.6	1.0	
No	522	280	53.6	1.6 (0.9-2.7)	0.13
Uses a toothbrush					
2 times/ daily or more	553	287	51.9	1.0	
1 time/daily or less	35	20	57.1	1.2 (0.6-2.5)	0.67
Uses dental floss					
2 times/ daily or more	144	59	41.0	1.00	
1 time/daily or less	444	248	55.9	1.8 (1.3-2.7)	<0.01
Uses a toothpick					
2 times/ daily or more	60	149	52.5	1.0	
1 time/daily or less	527	19	76.0	3.5 (1.3-9.0)	<0.01

In the multivariate analysis, the variables that showed a significant association with oral infection were: having a primary education or lower [OR=2.65; 95%CI=1.29-5.46; P<0.01]; last dental visit more than 6 months ago [OR=1.92; 95%CI=1.11-3.35; P=0.02]; flossing once a day or less [OR=2.47; 95%CI=1.35-4.52; P<0.01]; regular intake of alcoholic beverages [OR=2.82; 95%CI=1.63-4.87; P<0.01]; self-report of poor oral health [OR=2.16; 95%CI=1.29-4.09; P=0.03]; and received recommendation to see a dentist during pregnancy [OR=0.53; 95%CI=0.30-0.95; P=0.03).

## Discussion

This study comprehensively evaluated oral changes, encompassing both infectious and non-infectious conditions. While oral infections represent the most clinically significant findings, broader alterations in the oral cavity also contribute to the understanding of oral health and its impact on pregnancy outcomes.

Oral infections were significantly associated with lower education levels, corroborating findings from other studies (7,8). Vieira et al. (7) observed that pregnant women with low educational levels and periodontal diseases were more likely to experience premature births. Similarly, Chalvatzoglou et al. (9) concluded that higher education levels were associated with better oral hygiene practices, such as regular tooth brushing and flossing. Cheng et al. (10) further demonstrated a relationship between maternal health literacy and prenatal care, reinforcing the importance of integrating health literacy initiatives into maternal health programs.

Another significant factor associated with oral infections was last dental visit over six months ago. Cultural and personal beliefs regarding dental treatment during pregnancy often discourage women from seeking preventive care, despite its importance. This aligns with findings from a study conducted in Tehran (11), which demonstrated that educational interventions based on health belief models were effective in preventing oral diseases. Regular dental visits are critical for both prevention and health promotion; however, their frequency among pregnant women remains alarmingly low. In the United States, for instance, only 23-35% of pregnant women visit the dentist regularly during pregnancy (12). Similarly, a study conducted in Saudi Arabia (13) revealed limited dental visits among pregnant women, underscoring the global need to raise awareness and improve access to oral health care services during pregnancy.

Flossing habits also emerged as a significant factor. Pregnant women who flossed once a day or less showed a higher likelihood of oral infections. Previous research (13) revealed that 19.1% of pregnant women were unaware of the importance of flossing, highlighting the need for targeted oral health education during pregnancy.

This study found that 76.4% of pregnant women exhibited periodontal changes, with 15.8% presenting periodontitis and 68.4% gingivitis. Poor oral hygiene, as indicated by bacterial plaque and dental calculus, was observed in 66.3% of participants. These findings align with previous studies linking hormonal changes during pregnancy, such as increased progesterone and estrogen, to increased inflammation in oral tissues (14-16). These hormonal changes exacerbate the inflammatory response to dental plaque, compromising the immune response and increasing susceptibility to oral infections (17).

Hurjui et al. (18) emphasized that gingival tissue changes during pregnancy are associated with an exacerbated clinical inflammatory response. Additionally, endogenous and exogenous factors, such as diet, socioeconomic status, and behavioral habits, modulate the oral microbiota, potentially leading to pathological changes (19).

Alcohol consumption during pregnancy was another significant behavioral factor identified in this study. Denny et al. (20) reported that one in nine pregnant women regularly consumed alcohol, with a third of these women engaging in excessive drinking. Similarly, Kim (21) found that alcohol consumption was higher among pregnant women than among breastfeeding women, with poorer oral health outcomes among the latter. These findings underscore the importance of addressing alcohol consumption in public health policies and prenatal care programs, as they have significant implications for maternal and fetal health.

This study revealed that 55.4% of pregnant women did not receive a recommendation for a dental visit during pregnancy, highlighting the need for multidisciplinary integration in prenatal care. Most participants (89.0%) were unaware of the importance of prenatal dental care, consistent with findings from previous studies (22-27). Despite the longstanding literature on pregnancy and oral health, this topic remains relevant due to persistent knowledge gaps. Educational programs have proven effective in improving oral health behaviors, as demonstrated by studies where pregnant women who received guidance were more likely to seek dental care (28,29).

This study's cross-sectional design limited the establishment of temporal relationships between the identified associations. Additionally, the used of self-reported data leads to potential bias, although the inclusion of clinical oral health examinations strengthens the study's validity. Future research should explore longitudinal designs to better understand causal relationships and investigate broader environmental and physiological factors impacting oral health during pregnancy.

This study highlights the critical importance of oral health during pregnancy and its impact on maternal-fetal outcomes. The high prevalence of oral changes observed in the pregnant women analyzed underscored the need for targeted health promotion strategies, educational interventions, and the integration of dental care into prenatal services. Addressing these challenges through multidisciplinary approaches can reduce inequities in oral health and improve health outcomes for both mothers and their babies.
